# Isolation-by-Distance and Outbreeding Depression Are Sufficient to Drive Parapatric Speciation in the Absence of Environmental Influences

**DOI:** 10.1371/journal.pcbi.1000126

**Published:** 2008-07-25

**Authors:** Guy A. Hoelzer, Rich Drewes, Jeffrey Meier, René Doursat

**Affiliations:** 1 Department of Biology, University of Nevada, Reno, Nevada, United States of America; 2 Biomedical Engineering Program, University of Nevada, Reno, Nevada, United States of America; 3 Department of Mathematics and Statistics, University of Nevada, Reno, Nevada, United States of America; 4 Institut des Systèmes Complexes, CREA, CNRS, and Ecole Polytechnique, Paris, France; University of California San Diego, United States of America

## Abstract

A commonly held view in evolutionary biology is that *speciation* (the emergence of genetically distinct and reproductively incompatible subpopulations) is driven by external environmental constraints, such as localized barriers to dispersal or habitat-based variation in selection pressures. We have developed a spatially explicit model of a biological population to study the emergence of spatial and temporal patterns of genetic diversity *in the absence* of predetermined subpopulation boundaries. We propose a 2-D cellular automata model showing that an initially homogeneous population might spontaneously subdivide into reproductively incompatible species through sheer isolation-by-distance when the viability of offspring decreases as the genomes of parental gametes become increasingly different. This simple implementation of the Dobzhansky-Muller model provides the basis for assessing the process and completion of speciation, which is deemed to occur when there is complete postzygotic isolation between two subpopulations. The model shows an inherent tendency toward spatial self-organization, as has been the case with other spatially explicit models of evolution. A well-mixed version of the model exhibits a relatively stable and unimodal distribution of genetic differences as has been shown with previous models. A much more interesting pattern of temporal waves, however, emerges when the dispersal of individuals is limited to short distances. Each wave represents a subset of comparisons between members of emergent subpopulations diverging from one another, and a subset of these divergences proceeds to the point of speciation. The long-term persistence of diverging subpopulations is the essence of speciation in biological populations, so the rhythmic diversity waves that we have observed suggest an inherent disposition for a population experiencing isolation-by-distance to generate new species.

## Author Summary

A commonly held view in evolutionary biology is that new species form in response to environmental factors, such as habitat differences or barriers to individual movements that sever a population. We have developed a computer model, called EvoSpace, that illustrates how new species can emerge when a species range becomes very large compared with the dispersal distances of its individuals. This situation has been called isolation-by-distance because remote parts of the range can take different evolutionary paths even though there is no particular place where we would expect different populations to separate. When the extent of genetic difference between individuals is coupled with decreasing offspring viability (e.g., resulting from developmental problems), EvoSpace predicts that sharp spatial boundaries can emerge in arbitrary locations, separating subpopulations that occasionally persist long enough to become reproductively incompatible species. The model shows an inherent tendency toward spatial self-organization, in contrast with the traditional view of environmentally forced origins of new species. We think that isolation-by-distance is a common aspect of the evolutionary process and that spatial self-organization of gene pools may often facilitate the evolution of new species.

## Introduction

The most common framework for understanding the process of biological speciation is geographical. For example, instances of speciation are typically allocated among three categories based on the extent of geographical separation between the daughter species. *Allopatric speciation*, in which a species range becomes severed and leads to population fragments that are not linked by gene flow, has been viewed as the most common means of speciation [1]. This process is easy to understand, because the independence of evolutionary processes (mutation, drift, selection) in populations that no longer communicate with one another would inevitably lead to reproductive incompatibility between such populations given enough time in isolation. Genetic incompatibilities are thought to accumulate in isolated subpopulations as first described by Dobzhansky [2] and Muller [3], and allopatric speciation has been modeled based on these ideas [4],[5]. Not only does genetic isolation between subpopulations simplify genetic modeling of speciation, it is also relatively easy to observe the “fingerprints” of allopatric speciation in many instances, such as the endemism of terrestrial species on islands (e.g., Darwin's finches [6]).

The two other geographical categories of speciation involve divergence between subpopulations in the face of gene flow, and it has been less clear what the compelling “fingerprints” of these processes might look like when observed after the fact. In the second category, *parapatric speciation*, one species becomes two where the daughter species occupy contiguous ranges. This has most often been modeled as a consequence of habitat variation and divergent local adaptation by subpopulations e.g., [7]–[10]. *Sympatric speciation*, in which the ranges of the daughter species overlap, has similarly been modeled as a consequence of microhabitat variability and niche-partitioning e.g., [10]–[12]. Models of sympatric speciation suggest that the efficiency of specializing in the exploitation of discretely different resources can favor the formation of two species over the maintenance of a single generalist species.

A common theme among all three of these categories is that speciation is induced by divisive, external factors and that the inherent tendency of biological populations is to remain unified in the absence of these factors. In other words, the conventional wisdom is that it is the environment that tears species apart, and that in the absence of local dispersal barriers or environmental heterogeneity, biological populations tend to sustain functional and genetic cohesion. One well-known, but rare, situation where this view breaks down is in the case of ring species [13]–[15]. The range of a ring species extends around some sort of environmental obstacle until the two ends of the range meet. If one were to sample the gene pool starting at one end of the distribution moving around the obstacle to the other end, the gene pool would become increasingly different from the starting point with distance traveled, as expected given relatively short dispersal distances within species with large ranges (isolation-by-distance [16]–[20]). Where the two ends meet, however, individuals with the greatest genetic differences within the species come into contact. If they are so different that they do not or cannot mate with one another, these local groups appear to be different species. This produces an enigma if local matings happen all the way around the obstacle, because the directly incompatible ends of the range still remain indirectly connected by gene flow. It is hard to say whether ring species represent one or two species, but these instances illustrate the potential for functional decoherence (speciation) under isolation-by-distance. This possibility, without the presence of obstacles to dispersal, is the focus of this study.

Isolation-by-distance can also lead to symmetry-breaking in the distribution of genetic variation across the species range, leading to the emergence of discretely different and spatially segregated subpopulations [21]–[28]. The formation of distinct, but reproductively compatible, subpopulations typically precedes speciation, so the inherent tendency of subpopulation emergence under isolation-by-distance further suggests the potential for autonomous speciation when dispersal distance is short relative to the species range. In this paper, we describe a model of a spatially extended biological population (isolation-by-distance) in the absence of both obstacles to dispersal and environmental heterogeneity, which suggests that biological populations inherently and regularly tend to tear themselves into reproductively incompatible daughter species.

Most previous spatial models of speciation have assumed predetermined subdivisions (e.g., island model or stepping-stone model), habitat variation-inducing localized selection differences, or both [4], [5], [29]–[31]. These external factors impinging on a population model constrain or determine the resulting spatial patterning of the gene pool. In contrast, subdivision resulting from isolation-by-distance alone is an organic consequence of the system's dynamics. Because the only evolutionary forces assumed by our model are mutation, recombination, dispersal, and outbreeding depression, it serves as a proof of concept that internal population dynamics can generate spatial subdivision of a gene pool, even to the extent of parapatric speciation.

## Results

### Overview of the Model

We have implemented a generalized cellular automaton model of evolutionary processes, called EvoSpace. The simulated population was distributed across an *N*×*N* grid, where cells were either unoccupied or occupied by one individual. An individual contained genetic information in the form of a set of chromosomes and could migrate and mate with other individuals within a certain distance. A chromosome consisted of a string of characters from the set {A, C, G, T} representing the nucleotide bases. The number and length of chromosomes were the same for all individuals. During mating, an offspring was constructed by randomly selecting and combining two haploid genomes from the two diploid parents, possibly introducing random mutations in the process. Thus reproduction in our model was sexual, because genomes from two parents were combined to produce the offspring and the two genomes within a parent exhibited recombination through the independent assortment of chromosomes during gamete formation; but individuals were also hermaphrodites, as any adult could potentially mate with any other adult.

At every generation (time step in the model), migration, mating, and mutations created genetically distinct offspring, but resulted in only minor changes in the spatial structure of population genetic variation. First, individuals could randomly move on the grid world within bounds determined by a *dispersal distance*. Then, they could choose a mate within similar bounds. Finally, after mating, each offspring was placed in a random cell in the vicinity of one of the parents while the parents died, so that only one generation lived on the grid at each time step. The mean reproductive rate in the population was regulated each generation to buffer swings in population size resulting from a variety of factors, such as stochastic mortality (see below). This was achieved by randomly removing individuals or generating additional offspring, reflecting a constant carrying capacity of the environment. Simulations began with a population of genetically identical individuals, amounting to 80% of the grid cells (20% of locations remained empty space), and the system was allowed to evolve for hundreds to millions of generations.

For the experiments described in this paper, the habitat across the grid environment was homogeneous, so location did not influence the fitness of individuals. However, we introduced one important dependency: the offspring's *survival probability* was a decreasing function of the *genetic difference* between merging gametic genomes. Expressing the genetic difference between two chromosomes as a fraction between 0 (nucleotide identities at all positions of the DNA sequence were identical) and 1 (nucleotide identities at all positions of the DNA sequence were different), offspring resulting from gametes with a genetic difference greater than a threshold *θ* had zero survival probability (we used *θ* = 0.6 in this study; see Materials and Methods). Conversely, gametes with a genetic difference less than *θ*_0_ were 100% compatible (we used *θ*_0_ = 0.05 in this study). The negative relationship between gamete genomic difference and offspring viability was a simple representation of Dobzhansky-Muller reproductive incompatibility. Orr [4] reasoned that the Dobzhansky-Muller outbreeding depression function would decline exponentially, rather than linearly, but our linear function provided a conservative approximation in this context: it required diverging subpopulations to persist for a much longer period of time as they absorbed the demographic cost of decreasing viability of hybrid offspring. We also tested a nonlinear alternative in the form of a truncated Gaussian distribution with a peak at 1%, which roughly traced the decline in our linear function. Not only did this eliminate the potential for artifacts associated with the angles of our “broken stick” function, but it also imposed a slight amount of inbreeding depression for gametic genomes that were too similar. The results from the Gaussian outbreeding depression function were qualitatively the same as those reported here for the linear function.

The outbreeding depression function was central to the exploration of speciation in this model, as reproductive isolation between sexual species lies at the core of the concept of speciation. It was, in fact, the fundamental criterion embodied in the definition most commonly assumed in the context of evolutionary biology: the Biological Species Concept (BSC) [1]. Our rule for reproductive isolation between species was somewhat more restrictive than the BSC requires, because real species could be genetically compatible, but behaviorally or morphologically incompatible. For a comprehensive discussion of reproductive compatibility functions in speciation models, see [10].

### Isolation-by-Distance

**Figure 1:**
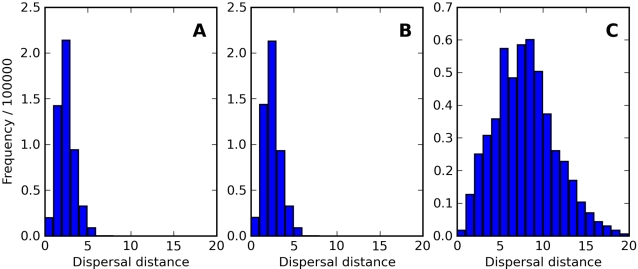
Histograms of Gene Flow Distances. Shown are histograms of the distances traveled, measured as simple Euclidean distance from grid cell center to grid cell center, for all chromosomes in the population within a single representative generation (step 200,000). Three typical simulations were conducted under the following selected simulation parameters: (A) movement distance *δ* = 1.5, outbreeding depression threshold *θ* = 0.6; (B) movement distance *δ* = 1.5, outbreeding depression disabled (*θ* = +∞); (C) movement distance *δ* = 5, outbreeding depression threshold *θ* = 0.6. For all graphs, target population size was 128,000, and grid size was 400×400. The movement distance parameter *δ* roughly defines the mean distance traveled by individuals in *each* of three phases within a generation (migration, mate selection, and offspring placement), so the net dispersal distance over a generation was usually greater than *δ* itself.

Gene flow distances in the model resulted from a combination of factors: the dispersal of individual agents, the location of female mates, and the settlement of offspring. The rules governing these behaviors of agents (see Materials and Methods) yielded a distribution of single generation gene flow distances that rarely exceeded six cells in our 400×400 spatial matrix when *δ* = 1.5, and 20 cells when *δ* = 5 (Figure 1).

**Figure 2:**
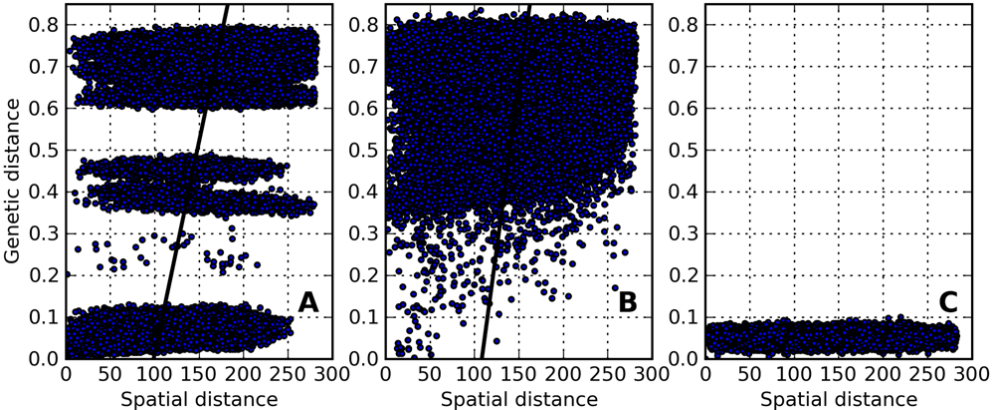
Scatterplots of Genetic Distance versus Spatial Distance. Shown are scatterplots of genetic distance versus Euclidean spatial distance between grid cell centers for 100,000 pairs of randomly selected individuals. Genetic distance was measured by comparing a randomly selected haploid from each chromosome, as described in the text. Simulation parameters were as in Figure 1 for A, B, and C. Plots 2A and 2B showed a tendency toward increasing genetic distance with increasing spatial distance, evidenced by the upward-sloping black trend line, but only the combination of isolation-by-distance and outbreeding depression (A) clearly showed multiple clusters representing distinctive genetic subpopulations. For the simulation run with large dispersal distance (C), genetic information was mixed across the landscape grid too quickly for local pockets of genetically distinct types to emerge, and the entire grid was filled with genetically similar, closely related individuals. Simple, linear regression lines are shown in (A) and (B). The regression line is not shown in (C) because the slope is not well defined by the data.

The shorter gene flow distances illustrated in Figure 1 generated a positive relationship between *geographic* and genetic distances, as described by the pattern view of isolation-by-distance (Figure 2). However, the distribution of points in Figure 2 seemed more informative than the slope of the regression line. Genetic surveys of real populations would not have the luxury of a sufficiently random sampling of such a large number of genomes, so it may be difficult to ascertain the distribution of points as effectively as was done here. Comparing the behavior of the model with and without the implementation of outbreeding depression showed very similar regression slopes, but very different patterns of point clustering, an important feature that could be easily missed with genetic survey data.

### Evolutionary Dynamics

**Figure 3:**
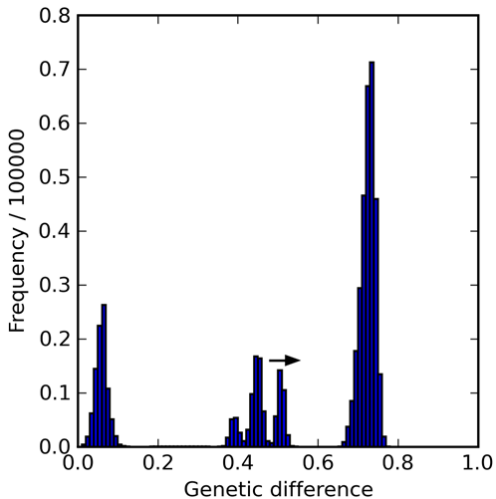
Example of a Mismatch Distribution. Shown are the frequencies of genetic distance classes between 500,000 randomly sampled pairs of haploid genomes. The multimodal distribution evident in this example indicated the existence of more than one distinctive gene pool in the population. The data here were drawn from generation 120,000 in a run where individuals contained *n* = 2 diploid chromosomes of *l* = 200 base pairs each, grid size was 400×400, population size was 128,000, maximum occupancy was 1 individual per cell, movement distance *δ* = 1.5, and mutation rate *μ* = 0.00005 per site per replication. Offspring viability dropped to 0 beyond a genetic distance of *θ* = 0.6. The presence of a distinct peak to the right of this threshold indicated the presence of reproductively incompatible populations. The peak to the far right never moved much beyond a genetic difference of 75%, the maximum value expected under the Jukes-Cantor mutation model [33]. The peak to the far left was also unmoving and remained centered near a genetic difference of *θ*_0_ = 0.05. This peak included all within-subpopulation comparisons. Peaks between these extremes always moved to the right; some eventually merged with the peak on the right, but most vanished first, indicating extinction of one or both subpopulations compared within the peak.

To assess the pattern of genetic diversity in our model system, we measured the genetic difference between two randomly selected, haploid gametes, as though these gametes were about to merge in fertilization and produce more or less viable offspring. We then analyzed this information with *mismatch distribution histograms* [32] to reveal the frequency distribution of genetic differences among the genomes in the population(s). The horizontal axis was the genetic difference, and the vertical axis showed the number of pairs of gametes found with that degree of genetic difference (Figure 3). In this plot, a population of genetically *random* individuals appeared as a single distinct peak at 0.75 (with a small standard deviation), because of the 25% probability that two bases were identical at any particular position of the DNA sequence. In the case of a population of genetically *identical* genomes—the starting condition for our simulations—the plot showed a single sharp peak at 0. As mutation led to genetic divergence, peaks traveled to the right in the mismatch distribution.

**Figure 4:**
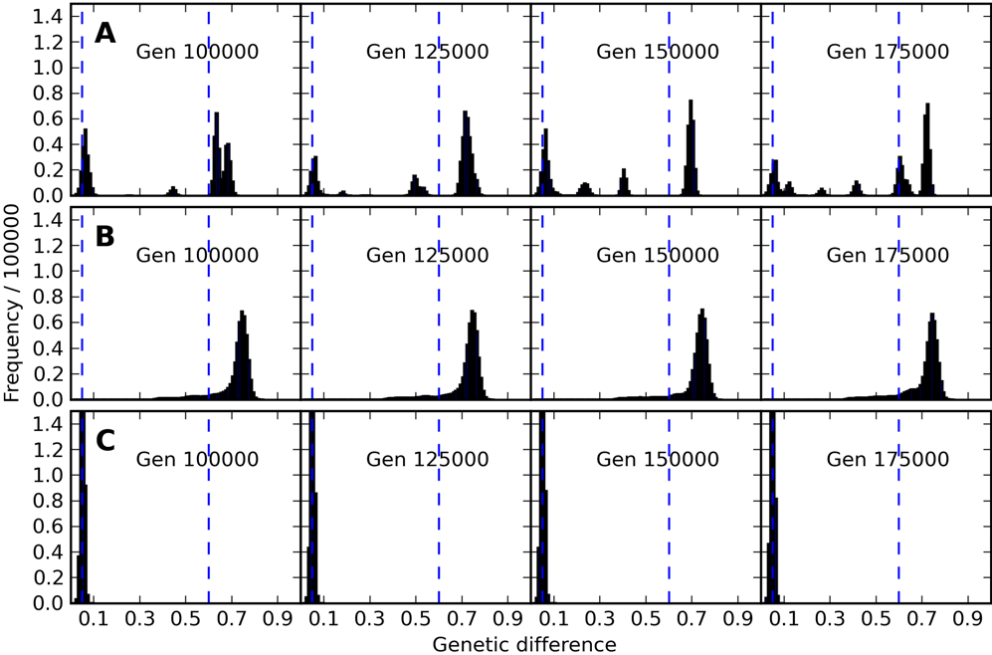
Time Series of Pair Similarity Histograms. Snapshots of frequency histograms of the genetic difference of randomly selected pairs of individuals from three runs, setting (A) low movement distance *δ* = 1.5 with outbreeding depression *θ* = 0.6, (B) low movement distance *δ* = 1.5 with no outbreeding depression, and (C) larger movement distance *δ* = 5 with *θ* = 0.6. Plots are shown horizontally for a sequence of four generations (100,000, 125,000, 150,000, and 175,000) within each of the conditions A, B, and C. The number of peaks does not correspond directly to the number of distinct genetic types in the population (see text for discussion of this important point). Video animations in MPEG format of the time course of the pair similarity histograms are available online in Video S1, Video S2, and Video S3. These animations are far more revealing of the fascinating time dynamics of these simulations than the small sequence of frames shown above.

Observing time-sequence movies of these mismatch distributions under different model conditions illustrated the spatiotemporal patterns of gain and loss of genetic diversity, especially as it revealed the origin and existence of distinct subpopulations (traveling waves along the distribution). The series of snapshots in Figure 4 provides a glimpse into the dynamics of these systems. When the population was effectively well mixed (Figure 4C; which is achieved here with *δ* = 5), genetic differences within the population did not grow far beyond the *θ*_0_ = 0.05 threshold, where outbreeding depression began to impact offspring viability. Under these conditions, the population mixed across the grid rapidly enough to remain a single, genetically coherent population. This was represented as a distinct and stable peak in the histogram at a genetic difference level of 5%. No pair of genomes was found with a genetic difference greater than 10% when *δ* = 5. All aspects of the model's behavior described here were repeatable for different runs of the model under the same conditions.

With *δ* = 1.5, but in the absence of outbreeding depression (Figure 4B), the single initial peak on the histogram centered on 0 spread and moved to the right as mutation created genetic variation. When the space was large enough, this primary peak became centered on a genetic difference of 75%, the maximum expected under the Jukes-Cantor mutation model [33]. For certain combinations of grid size (sufficiently large), dispersal distance (sufficiently short), and mutation rate, however, additional dynamical patterns emerged. We were particularly interested in tracking the diversity waves described by Rogers and Harpending [32]. Indeed, small peaks arose at low levels of genetic difference (left side of the mismatch distribution), moved to the right, and often persisted long enough to merge with the primary peak. This observation is consistent with previous findings on spatial self-organization under isolation-by-distance in the absence of outbreeding depression [21], [22], [34]–[40].

The patterns that we detected in intraspecific dynamics were greatly enhanced by combining the outbreeding depression function with the shorter dispersal distance (Figure 4A). Along with sharpening the degree of spatial organization that emerged (see next section), outbreeding depression strongly increased the amplitude and separation of the secondary peaks appearing in the mismatch distributions. It also firmly established a large peak centered on *θ*_0_ (5% in this case), representing all within-subpopulation comparisons. In effect, each emergent subpopulation functioned like a single panmictic population. The traveling waves peeled off this peak as existing subpopulations divided. As these peaks moved to the right of *θ*_0_, the subpopulations being compared experienced an increasingly stringent demographic disadvantage, because when individuals from diverging subpopulations mated with each other, their offspring were decreasingly viable. Divergence continued as the demographic cost of outbreeding increased. Nevertheless, sometimes a peak became established to the right of *θ*. This represented a set of comparisons between gametes from *reproductively isolated* subpopulations, since their hybrid offspring could not be viable anymore. In summary, we interpret the traveling waves to reflect discrete genetic subpopulations, *new species* when they persisted past *θ* = 0.6, emerging through the spatial microevolutionary dynamics within a population.

We have further found that the development of new, stable peaks to the right of *θ* (new species, we argue) was quite sensitive to the interrelationships of the spatial scale of the simulation (grid size and dispersal distance), the mutation rate, and other factors. For example, if the mutation rate was too high, overall genetic diversity increased rapidly until it was too hard to find viable mating pairs within the mating neighborhood and the whole population went extinct. If the mutation rate was too low, the degree of genetic difference generated between subpopulations did not reach the threshold of speciation before at least one of the subpopulations went extinct. We are working now to examine systematically the likelihood that biological populations would evolve within the region of phase space associated with these interesting dynamics.

### Spatial Self-Organization

**Figure 5:**
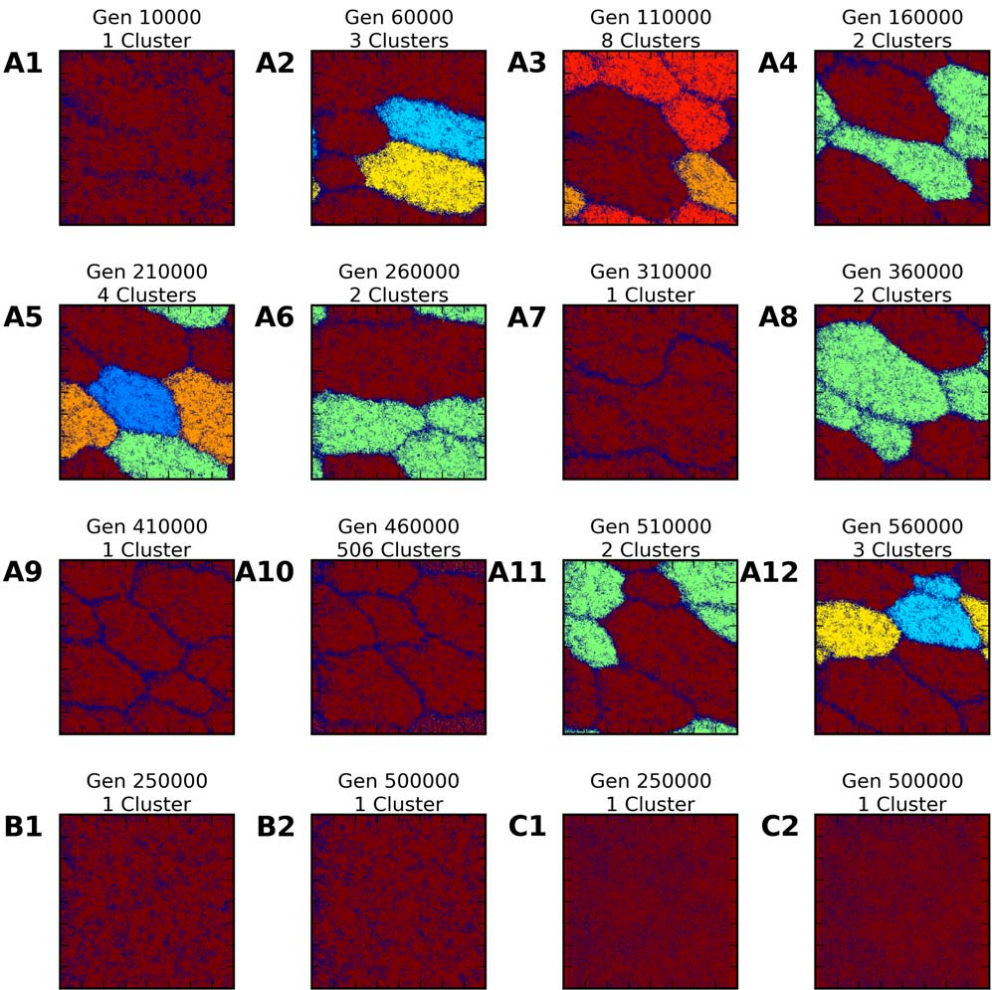
False-Color Depiction of Genetic Clustering on the World Grid. Dark blue represents unoccupied cells or cells from which genomes were sampled that were not connected to any cluster. Each other color represents a set of gametes with genome sequences that are identical at more than 40% of their nucleotide sites, created according to the algorithm described in the text. Gametes colored differently have genomes that are identical at 40% or less of their nucleotide sites. Using this threshold in combination with *θ* = 0.6 helps identify different species with different colors. Note that (1) colors were assigned anew in each plot, so particular colors do not track the same lineage across plots, (2) the clustering algorithm is probabilistic because it is computationally expensive to compare every individual with every other individual for determination of genetic difference, and (3) some clusters are too small to discern in these plots. Plots (A1) through (A12) depict snapshots of the clustering state at 12 different generations during the simulation run with low movement distance of *δ* = 1.5 and outbreeding depression threshold *θ* = 0.6, the same simulation shown in the (A) portion of earlier figures. Also following previous figures, plots (B1) and (B2) were generated under a low movement distance of *δ* = 1.5 without outbreeding depression. This model shows no evidence of clustering at the difference threshold of 0.6 for two representative generations (250,000 and 500,000), nor for any other generations examined (not shown here). The (C) plots, for a simulation run with a larger movement distance of *δ* = 5 and outbreeding depression enabled at a threshold of *θ* = 0.6, presents a similar lack of clustering.

The mismatch distribution provided good insight into the existence of distinct, internally homogenous subpopulations, but it did not demonstrate whether clusters were spatially segregated on the lattice or show where they were located. Two other analytical tools, isolation-by-distance scatterplots (Figure 2) and *genetic cluster plots* (Figure 5), were useful in examining the *spatial* clustering of distinct subpopulations.

Genetic cluster plots were obtained by grouping sets of individuals for which all genetic distance relations were lower than a given level and displaying those groups in different colors (Figure 5). These plots clearly illustrated the spatial self-organization that emerged in our model. Visual examination indicated that the genetically homogeneous subpopulations revealed by the histogram plots also formed distinct spatial clusters that occupied coherent, non-overlapping regions of the lattice. The borders between these regions tended to be unoccupied, or occupied with hybrid genomes that were not genetically similar enough to any neighboring region to be classified with them.

Spatial plots such as isolation-by-distance and genetic clustering also demonstrated the strong sharpening effect of outbreeding depression. The intrinsic tendency of the grid world toward spatial order was greatly enhanced by introducing dependence of viability on similarity. It could be said that outbreeding depression played a negative feedback role analogous to long-range inhibition in morphogenetic reaction-diffusion processes [41],[42]. In this analogy, the combination of mating and gene flow played the positive feedback role of short-range activation. Together, these effects contributed to the spontaneous formation of “spots” by encouraging neighboring elements to be similar and, at the same time, distant elements to be different. In a sense, our model represents “evolutionary pattern formation” at the scale of populations of organisms, instead of morphogenetic pattern formation at the scale of tissues of cells.

## Discussion

Spatially explicit computational models of evolution are a relatively recent development made possible by the rapid rise in the power of computing hardware, although the earliest studies date back to the 1970s [21]. A pervasive, and perhaps universal, behavior exhibited by these models is the tendency for heritable variation to become spatially segregated in a process of self-organization e.g., [22], [27], [34]–[40]. Our model also exhibited this phenomenon, as we expected. It is important to recognize this inherent tendency for spatial diversification and the natural ways in which this would facilitate speciation, even if the model presented here does not represent a complete description of any particular speciation event. It should also be noted that the spatial dynamics of speciation in the absence of dispersal barriers is significantly more complicated than the issue of how Dobzhansky-Muller incompatibilities accumulate in isolated populations [4],[5], although they share the endpoint of completed speciation. Connected populations can become disjunct, creating the opportunity for allopatric speciation, but sexual recombination and dispersal make spatially-extended genetic networks the essence of sexual populations. This is also the basis of EvoSpace, which is used here to provide a spatially connected context for studying the evolution of reproductive incompatibilities among emergent (not assumed) subpopulations.

The dominant geographic paradigm for classifying modes of speciation recognizes three general categories: allopatric, parapatric, and sympatric speciation. The allopatric mode has long been widely thought to represent the most commonly realized mode [43]. The sympatric mode of speciation has also received much attention over the past 50 years or so, although it has long been considered controversial [43]. Recently, however, it seems that the potential for sympatric speciation has become more widely appreciated and some compelling empirical examples have come to light e.g., [44]. Parapatric speciation has received less attention (but see [10]), perhaps because it is something of a hybrid between the other two. It requires speciation in the face of gene flow, which is the same hurdle that must be overcome in achieving sympatric speciation, yet it involves subpopulations and sibling species that have essentially non-overlapping ranges, as in allopatric speciation. Models of parapatric speciation have typically involved environmental variation applying divergent selection pressures to different parts of the species range, with that reinforcing selection favoring positive assortative mating within the hybrid zone completes the speciation process [7]–[10]. In contrast, our model can illustrate a process of parapatric speciation *in the absence* of environmental variation and preferential mating. By including functional outbreeding depression, we have invoked an endogenous kind of selection that is independent of the external environment. Thus this is a model of speciation through the spatial *self-organization* of the gene pool.

Our model is similar to the neutral model developed by Gavrilets and colleagues, although there are fundamental differences [29]–[31]. Both approaches account for the entire process of speciation, from genetic homogeneity to reproductive isolation, but a key difference is represented in the geographic assumptions of the models. The neutral models of Gavrilets [29]–[31] assume a discretely subdivided population, connected by migration, at the start. Our model assumes a population with absolutely no predetermined subdivision or barriers that would divide subpopulations, yet it is able to self-organize into discrete subpopulations that can then evolve reproductive isolation. It is interesting that these two kinds of models behave in similar ways. For example, both models show that speciation is possible even in the presence of gene flow, and both models show that local adaptation is not necessary to generate reproductive isolation. Another important difference between these models regards the shape of the outbreeding depression function. Gavrilets assumes a step-shaped function where offspring viability is either 0 or 1, depending on the extent of genetic difference between gametes. In this way, reproductive isolation between individuals happens as a byproduct of a single mutation: the one that pushes the genetic difference between two individuals over the reproductive incompatibility threshold. In our model, reproductive incompatibility accrues by degree, requiring subpopulations gradually to take on the increasing demographic cost of more failed reproductive opportunities as the speciation process unfolds.

Several papers by Sayama, Bar-Yam, and colleagues [27],[28],[34],[35] have emphasized the role of spatial dynamics in the spatial patterning of gene pools, but the model that they have developed does not include a mutation process and assumes an artificial fitness function. Their model envisions two compatible sets of alleles across loci, and mixing alleles across these sets is assumed to result in decreased fitness. While there is heuristic value in observing how allelic incompatibilities sort themselves in space, it is hard to imagine how allelic variation with these features might evolve in the first place. This combination of assumptions results in a population with two distinct genotypes, each assumed to be internally compatible, with clumped distributions in space. While spatial self-organization is evident in this model, demographic stochasticity would ensure that it ultimately evolves to complete homogeneity in the absence of mutation. EvoSpace allows genetic incompatibilities to arise organically through mutation and lineage proliferation within the rules of the model. The occurrence of mutation within EvoSpace also allows the population at large to accumulate and organize genetic diversity to an extent that is sustainable under the model.

Under the set of parameter values explored here, our model suggests that mutation alone can drive genetic divergence between subpopulations to the point of overwhelming the constraints imposed by (limited) gene pool mixing and outbreeding depression under isolation-by-distance. The emergence of sharp boundaries between genetic subpopulations is consistent with previous computational models incorporating isolation-by-distance [22], [27], [34]–[40], although the natural tendency for spatial self-organization in population genetics has not yet been fully appreciated by the community of population geneticists. Indeed, there has been a long-standing confusion in the literature between the notions of isolation-by-distance as a process or as a pattern [45]. Sewall Wright [16] originally conceived of isolation-by-distance as a model of the evolutionary process in which

there is complete continuity of distribution, but interbreeding is restricted to small distances by the occurrence of only short range means of dispersal. Remote populations may become differentiated merely from *isolation by distance.* (original emphasis)

The presumed pattern of isolation-by-distance is a smooth, monotonically increasing relationship between geographic distance and genetic distance [17]–[20], which does not anticipate sharp transitional boundaries between internally homogeneous, divergent subpopulations. This expectation was based on mathematical models of Wright's [16] process view of isolation-by-distance that were not able to predict emergent population substructure because they relied on mean field approximations of spatial context that did not represent spatial configurations. Therefore, we advocate a return to Wright's original view of isolation-by-distance as part of the evolutionary process characterized by relatively short dispersal distances within an extensive population range and open-mindedness to the possibility that isolation-by-distance alone can result in the emergence of spatially bounded subpopulation structure.

The model of evolutionary genetics presented here is a very simple and generic one. It does not depend on idiosyncratic forms of selection or particular population structures. Instead, it is based on fundamental and common building blocks of biological populations (chromosomes and sexual individuals) that are stochastically affected by mutation, mortality, reproductive success, and dispersal. The interesting behavior of the model emerges dynamically due to the constraints of isolation-by-distance and outbreeding depression. Therefore, we conjecture that the tendency for spatial self-organization and parapatric speciation may occur universally in biological populations. This is not a claim that our model is the exclusively correct model of speciation; rather, we are suggesting that the dynamic of diversification illustrated by our model may exist even under conditions that suppress the realization of emergent population substructure, such as great dispersal distances in relatively small species ranges. We expect that the inherent tendency for spatial diversification amplifies the effects of environmental heterogeneities, and we plan to explore this interaction with further developments of EvoSpace. Thus we do not deny that habitat variation and dispersal barriers can play important roles in instances of speciation, but we think that the inherent dynamic identified here may generally drive diversification/speciation in a way that is molded to these external constraints.

In conclusion, our model reveals an aspect of intraspecific evolutionary dynamics that emerges when dispersal distances are sufficiently short relative to a species range. Localized subpopulations regularly form and diverge from one another while maintaining their identities as clusters of genetically similar individuals. Subpopulations that grow so large as to embody too much genetic diversity tend to subdivide through spatial segregation, just as the original population does in our model. If the degree of outbreeding depression grows as gametes' genomes become increasingly different, the pattern of genetic and spatial population subdivision becomes better defined, and some subpopulations can diverge to the point where they complete a process of parapatric speciation. The behavior of our model suggests that spatially extended populations regularly generate new subpopulations, each of which takes a path of genetic divergence with the potential of becoming a new species. Most of these embryonic subspecies become extinct before emerging as reproductively independent species, but the internal dynamic of this model constantly potentiates the production of new species.

## Materials and Methods

### The World Grid and Dispersal Across It

The simulated world is an *N*×*N* grid that wraps around north-south and east-west, creating a torus. Typical grid sizes are 100×100 to 500×500 cells (see samples in Figure 5). Smaller grids run faster and consume less memory, allowing flexible parameter exploration, while larger grids allow investigation of larger-scale spatial isolation effects. Each grid cell is constrained to contain a maximum of *m* occupants. For the results reported here, we use *m* = 1 (i.e., 0 or 1 occupant per cell). Grid coordinates are Cartesian pairs **r** = (*x*, *y*), and distances between cells are simple Euclidean distances from the cell centers. Thus each cell has exactly four neighbors at distance 1, four more neighbors at distance √2, and so on. A simulation parameter *δ* correlates with the distance within which individuals can move, mate, or appear in a single generation. As the value of this parameter increases, so too does the mobility of the individuals within the grid world. Movements of individual genomes, carried by either diploid individuals or haploid gametes, occur in three stages: *migration*, *mating*, and *offspring placement*. (a) To begin with, each individual migrates to another site randomly determined by the combination of a Gaussian and a uniform probability distribution. Denoting by **r** the starting location of an individual, its new location **r**′ after migration is calculated in two steps. First, a distance *d* is drawn from a Gaussian distribution of mean and standard deviation *δ*: *G_δ_*(*d*) = *κ* exp[−(*d*−*δ*)^2^/*δ* ^2^], where *κ* is a normalization coefficient. Second, the location **r**′ is drawn from a uniform distribution within a disc *D* of radius *d* centered around **r**: *P_mig_*(**r**′|**r**) = *D_d_*(**r**′−**r**), where *D_d_*(**u**) = 1/*πd* ^2^ if *||* **u** *||*≤*d* and 0 otherwise. (Additionally, **r**′ is corrected to fall on the nearest integer grid location.) (b) Then, each individual is considered in turn to act as the “father” and sends a gamete to a potential “mother” **r**″ at a location chosen uniformly randomly in a circle of radius *δ* centered around the father's location **r**′: *P_mat_*(**r**″|**r**′) = *D_δ_*(**r**″−**r**′). (c) Finally, a newly formed offspring (see next section) settles into a grid cell **r** *′′′* drawn uniformly randomly in a circle of radius 2*δ* around its mother's position *P_off_*(**r** *′′′*|**r**″) = *D_2δ_*(**r** *′′′*−**r**″).

### Sexual Reproduction and Offspring Viability

**Figure 6:**
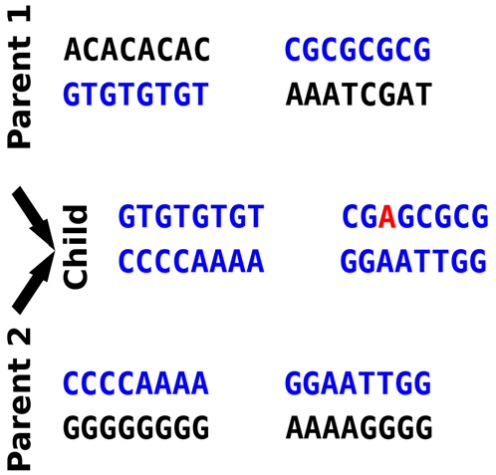
Virtual Genomics and Sexual Reproduction. Individuals are diploid hermaphrodites, in which haploid genomes consist of *n* = 2 chromosomes containing *l* = 200 nucleotide bases A, C, G, and T (only *l* = 8 bases per chromosome shown in this diagram). Each parent produces a haploid gamete by randomly selecting one of the chromosomes from each diploid pair (blue portions of the parent). When producing a gamete, some random point mutations may also occur with a low probability *μ* per site. One such mutation, from base C to base A, is depicted in red.

Each individual contains its personal genetic information as a diploid set of chromosomes. A chromosome consists of a string of characters that can take one of four values representing the nucleotide bases (A, C, G, or T). The number *n* and length *l* (number of base pairs) of chromosomes are the same for all individuals and are both set at the start of a simulation; *n* = 2 and *l* = 200 for all results presented here. Reproduction in our current model is sexual, because genomes from two parents are combined to produce the offspring, but individuals are also hermaphrodites able to function as either the male or female in a sexual encounter. An offspring is constructed from two parents as follows. Each parent produces a haploid gamete by randomly selecting one of the chromosomes from each diploid pair (Figure 6). These haploid genomes are combined in the offspring to produce a diploid individual. Thus we have not allowed for crossing over within chromosomes, but non-homologous chromosomes assort independently during sexual recombination. During the transcription from each parent to its gamete, some random mutations in the form of single base substitutions are introduced according to a uniform and constant probability *μ* defined at the start of the simulation; *μ* = 0.00005 mutations/site for results given in this paper.

**Figure 7:**
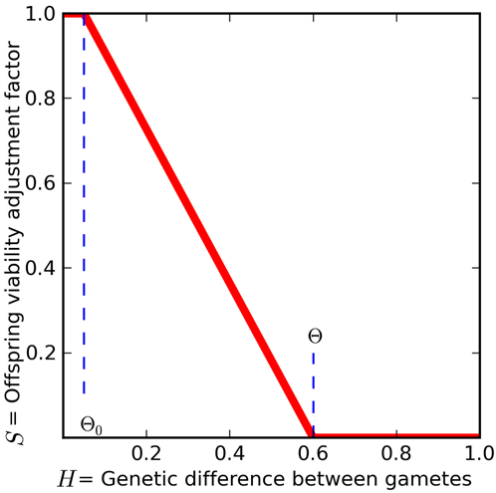
Offspring Survival Probability *S* as a Function of Genetic Difference *H*. For all the results presented in this paper, viability is not reduced if gametic genomes differ only by *θ*_0_ = 0.05 or less (this is an adjustable parameter in the model). If gamete genomes differ by more than 5%, offspring survival probability is reduced linearly to eventually reach 0 for a threshold amount *θ* of genetic difference (*θ* = 0.6 in this graph and in all simulations presented here).

The *genetic difference H* between the two merging gametic chromosomes in a diploid pair is expressed as the fraction (between 0 and 1) of all the base pairs containing different bases: *H* = *B/nl*. Thus *H* = 0 means that all bases in the child's gametes #1 and #2 are identical at every position, and *H* = 1 means they all differ at every position. The offspring's *survival probability S* can be set to be dependent on this genetic difference, according to the curve in Figure 7, at the beginning of a run. Given two threshold values of genetic difference *θ*_0_ and *θ*, such that 0≤*θ*_0_≤*θ*≤1, we set *S* = 1 for *H*≤*θ*_0_, *S* = 0 for *H*≥*θ*, and *S* = (*θ*–*H*)/(*θ*–*θ*_0_) for *θ*_0_<*H*<*θ*. Thus offspring composed of gametes with a genetic difference *H* greater than *θ* are nonviable and immediately removed from the grid at birth. In general, two random genome sequences are expected to have a genetic difference *H* = 0.75, since each nucleotide position would have a 25% chance of being occupied by the same base [33].

### Computation of Generations

The simulation begins at generation 0 with a population of genetically identical individuals. The initial population randomly fills a predetermined fraction of the maximum grid occupancy. For the experiments described in this paper, the grid occupancy fraction is 80%. For example, a grid of 400×400 = 160,000 cells starts with 128,000 individuals.

The creation of the next generation for the simulation is a three-phase process. (a) First, each individual *migrates* to a random cell **r**, according to the probability *G_δ_* described above. (b) Then, individuals *reproduce*. The algorithm iteratively considers each member of the population once to be the “father” in a mating. For each father, a list of potential mates is created from those individuals in all cells within a radius *δ* of the father, and one of those individuals is randomly selected to be the “mother.” Mating then proceeds as described above. The number of potential offspring resulting from each mating is given by a Poisson distribution with mean 1. (c) Finally, each offspring is placed in a random cell within a radius 2*δ* of the mother. If no cell within this range has room for the offspring according to the maximum per-cell occupancy *m*, then some or all offspring of this mating could be lost. No parents survive into the next generation, so overcrowding can be the result only of offspring from matings that have already taken place during earlier processing of the current generation. Since each individual is the father in exactly one mating, and the mother in an average of one mating, and each mating has an average of one offspring, the population size should stay about the same from generation to generation. Certain factors, however, could result in the population size shifting from the target size, which is addressed in the next phase.

In a supplementary step, *adjustment*, the algorithm makes an effort to keep the population close to the initial target population size. This may require additional “make-up” births from randomly selected parent pairs, if offspring viability reduced the population size due to high genetic difference of parents, or if localized overcrowding resulted in the loss of some offspring. More rarely, if the population ends up above the target level, the algorithm randomly selects individuals for culling.

### Genetic Distance Computation

Given any two diploid individuals on the grid, we draw one haploid genome from each individual by randomly selecting one chromosome in each diploid pair they contain (a process identical to constructing a haploid gamete for that individual). The genetic distance (as plotted in Figure 1) is then defined by counting the number of mismatched base pairs between these two haploid genomes and dividing by the total number of base pairs. Due to the random assortment of chromosomes, this quantity is not necessarily the same every time it is computed for a given pair of individuals. We also use the term “distance” without verification that the triangle inequality holds.

### Mismatch Distributions

A mismatch distribution reveals the shape of genetic diversity in a sample through pairwise comparisons of genetic differences, which are displayed in a frequency histogram [32] (Figure 3 and Figure 4). The number of peaks on the histogram does not directly correspond to the number of genetically self-similar subpopulations. For example, in the case of a population with three genetically distinct groups, A, B, and C, where A and B have an average genetic difference of 60%, A and C of 40%, and B and C of 20%, the histogram would show these three peaks plus one centered on *θ*_0_ (0.05 for the results presented here) representing the within subpopulation comparisons. However, if clusters B and C also happened to be 0.4 distant, two peaks would be superimposed and would obscure the number of subpopulations. A peak typically represents a set of comparisons between two subpopulations defining the degree of divergence between the subpopulations. The peaks move to the right in the mismatch distribution as long as both subpopulations persist and evolve along different trajectories, and the peaks stop moving when divergence hits the maximum value of 75% expected under the Jukes-Cantor mutation model [33].

### Isolation-by-Distance (IBD) Scatterplots

Scatterplots with geographic distance on the *x*-axis and genetic distance on the *y*-axis (Figure 2) are commonly presented as a way to examine isolation-by-distance in data from spatial genetic surveys. It is expected that shorter dispersal distances will yield a steeper slope for this relationship, although the relationship may not be linear [46]. An effectively well-mixed population should show no relationship between geographic and genetic distances, because thorough mixing would randomize location with respect to genotype.

### Genetic Cluster Plots

A genetic cluster plot (Figure 5) is constructed by randomly selecting pairs of haploid genomes and computing their genetic distance, similarly to the mismatch distributions, then only retaining pairs that are distant below a certain level set by the user. When a sufficient number of pairs with low distances have been gathered, we build a nondirected graph that contains the individuals as nodes and edges representing genetic distances smaller than the threshold. By analyzing this graph we can then identify regions of self-connected clusters. Since it is not computationally feasible to examine all pairs of individuals, the clustering might depend on the random selection of pairs of individuals (i.e., some clusters that were not connected in one graph construction might be connected in another). We have found, however, that repeated application of our clustering algorithm with different random seeds (leading to different pairs being examined) leads to qualitatively identical results. On the other hand, reducing the distance threshold has the expected effect of connecting formerly disconnected genetic clusters.

## Supporting Information

Video S1 Short dispersal distance with oubreeding depression.
(7.38 MB MPG)

Video S2 Short dispersal distance without outbreeding depression
(7.23 MB MPG)

Video S3 Longer dispersal distance without outbreeding depression
(6.15 MB MPG)
